# Screening the yeast genome for energetic metabolism pathways involved in a phenotypic response to the anti-cancer agent 3-bromopyruvate

**DOI:** 10.18632/oncotarget.7174

**Published:** 2016-02-03

**Authors:** Paweł Lis, Paweł Jurkiewicz, Magdalena Cal-Bąkowska, Young H. Ko, Peter L. Pedersen, Andre Goffeau, Stanisław Ułaszewski

**Affiliations:** ^1^ Department of Genetics, Institute of Genetics and Microbiology, University of Wrocław, Wrocław, Poland; ^2^ KoDiscovery LLC, UM BioPark, Innovation Center, Baltimore, MD, USA; ^3^ Departments of Biological Chemistry and Oncology, Sydney Kimmel Comprehensive Cancer Center and Center for Obesity Research and Metabolism, John Hopkins University School of Medicine, Baltimore, MD, USA; ^4^ Unité de Biochimie Physiologique, Institut des Sciences de la Vie, Université Catholique de Louvain-la-Neuve, Louvain-la-Neuve, Belgium

**Keywords:** 3-bromopyruvate (3-BP), Saccharomyces cerevisiae, energetic metabolism, genomic screen, Whi2

## Abstract

In this study the detailed characteristic of the anti-cancer agent 3-bromopyruvate (3-BP) activity in the yeast *Saccharomyces cerevisiae* model is described, with the emphasis on its influence on energetic metabolism of the cell. It shows that 3-BP toxicity in yeast is strain-dependent and influenced by the glucose-repression system. Its toxic effect is mainly due to the rapid depletion of intracellular ATP. Moreover, lack of the Whi2p phosphatase results in strongly increased sensitivity of yeast cells to 3-BP, possibly due to the non-functional system of mitophagy of damaged mitochondria through the Ras-cAMP-PKA pathway. Single deletions of genes encoding glycolytic enzymes, the TCA cycle enzymes and mitochondrial carriers result in multiple effects after 3-BP treatment. However, it can be concluded that activity of the pentose phosphate pathway is necessary to prevent the toxicity of 3-BP, probably due to the fact that large amounts of NADPH are produced by this pathway, ensuring the reducing force needed for glutathione reduction, crucial to cope with the oxidative stress. Moreover, single deletions of genes encoding the TCA cycle enzymes and mitochondrial carriers generally cause sensitivity to 3-BP, while totally inactive mitochondrial respiration in the rho^0^ mutant resulted in increased resistance to 3-BP.

## INTRODUCTION

Research using yeast *Saccharomyces cerevisiae* has provided much information concerning the mechanisms of cellular metabolic processes, cell cycle regulation, recombination, replication and repair of DNA as well as the cell death mechanisms, protein folding and biogenesis of organelles [1]. It is predicted that almost half of all yeast genes (about 3000) have structural or functional homologues in the human genome [2]. Further, yeast genome was the first eukaryotic genome to be fully sequenced [3], which accelerated the development of new holistic research methods, such as usage of deletion and overexpression libraries, two-hybrid analysis and microarrays [4]. Yeast model has proven its usefulness as a first-line tool in discovery of mechanisms of basic processes engaged in pathogenesis thanks to the conserved protein interaction networks. The direct contribution is the basic knowledge on the cell cycle regulation [5]. The discovery and analysis of apoptotic processes in yeast allowed better understanding of the molecular mechanisms underlying apoptosis-related diseases [6]. Heterologous expression of human proteins in yeast cells allowed understanding of the functions of many disease-related proteins leading to the identification of the disease progression mechanisms. Research using yeast has also provided relevant information regarding molecular basis of cancer, resulting in the identification of new therapeutic targets. There are certain phenotypic characteristics of yeast rendering them a perfect model system to investigate human diseases, as many of the basic cellular processes are well conserved [7]. New anticancer drugs can be tested on yeast model using specific mutant strains having some phenotype characteristics similar to cancer cells (such as faulty cell cycle control). For example, the molecular mechanisms of topoisomerase II inhibitors, used as anticancer drugs, were identified using 70 yeast strains with defective checkpoint control and faulty DNA repair [8]. Usage of proper mutants also allows testing for sensitivity, resistance mechanisms to new drugs as well as synergistic interactions with other drugs [9].

Like cancer cells, fermenting yeast exhibit overexpression of glycolytic enzymes in response to high glucose availability. Activity of the key enzymes (i.e., hexokinase, phosphofructokinase and pyruvate kinase) is highly increased. When growing on glucose, predominantly expressed isoform of hexokinase is HK II which is insensitive to inhibition by its product [10]. Furthermore, the first isoform of pyruvate kinase (Pyk1p), which is more susceptible to allosteric activation by fructose 1,6-bisohosphate, is strongly expressed. Because of high cytoplasmic metabolism of pyruvate only a small amount is oxidized and utilized in the Krebs cycle [11]. Together with decreased rate of TCA, the enzymatic activities of aconitase, isocitrate dehydrogenase and malate dehydrogenase are diminished [12]. Homologues of mammalian PDK (pyruvate dehydrogenase kinase) enzymes (Pdhk) and corresponding phosphatases (Pdp) are present in yeast [13]. The activity of yeast pyruvate dehydrogenase is regulated by its phosphorylation. The inactive dephosphorylated form is present mainly in cells growing on nonfermentable carbon sources, thus generating ATP through oxidative phosphorylation [14]. The pyruvate dehydrogenase complex is also regulated through its E3 subunit (lipoamide dehydrogenase), which expression is decreased during fermentative metabolism [15]. Lactate dehydrogenase occurs in yeast as two isoforms localized in the inner mitochondrial membrane. These enzymes irreversibly convert lactate into pyruvate and take part in the respiratory chain. The cytosolic form of lactate dehydrogenase is still unknown in yeast [16]. In yeast the direction of pyruvate metabolism in cytosol is mainly dependent on pyruvate decarboxylase (Pdc) activity [17]. Pdc converts pyruvate into acetaldehyde which is then oxidized in mitochondria [18] or reduced to ethanol by alcohol dehydrogenase (Adh). Although the metabolism of pyruvate in yeast and mammalian cells is not identical, still the general mechanism is the same – increased flux of this pathway diminishes the availability of substrates for aerobic metabolism, as it is in the case of hyperactive LDH in cancer cells. All these suggest that during intense fermentation, Pdc activation is strictly connected to the repression of mitochondrial respiration [19].

Metabolic similarities between fermenting yeast cells and tumor cells allow the research concerning metabolism-targeting anti-cancer drugs, e.g., 3-bromopyruvate. 3-BP is a structural analog of pyruvic acid and is highly reactive - it shows strong alkylating properties toward proteins. The pyruvic chain covalently binds to the cysteine and histidine residues changing the protein conformation and activity. 3-BP is also a putative inhibitor of all reactions involving pyruvate. Additionally, it was shown to inhibit hexokinase II [20]. During the tests on rats, mice and rabbits 3-BP exhibited high anticancer activity [21, 22]. All of the rats carrying a hepatoma which were treated with 3-BP, were fully cured with no recurrence. About 70% of cancer cells were killed after one administration and after 4 weeks of treatment the rats fully recovered [22].

The mechanism of selective anticancer effect of 3-BP is still not fully clarified. 3-BP probably inhibits glycolysis by acting on enzymes catalyzing reactions involving pyruvate (i.e., LDH, PC and PDH) leading to ATP depletion. 3-BP is also thought to cause inhibition of hexokinase II, which may lead to apoptosis of the cell [20, 22, 23]. Moreover, it was shown in vitro that 3-BP is able to alkylate glyceraldehyde 3-phosphate dehydrogenase (GAPDH) leading to its inhibition [24]. It is also assumed that 3-BP causes generation of reactive oxygen species (ROS) which are highly toxic for the cell [25]. It was shown that depletion of the intracellular glutathione strongly increased sensitivity of yeast cells to 3-BP, which suggests that 3-BP causes oxidative stress and may deplete the pool of reduced glutathione itself [26], which has also been shown in human erythrocytes [27]. The specific activity of 3-BP toward cancer cells may be also due to the selective uptake into the cells. 3-BP was shown to enter the cells through the Monocarboxylate Transporters (MCT), transporters of lactate and pyruvate. Research in breast cancer cells shown that butyrate, which induces the expression of monocarboxylate transporter MCT4, causes increased sensitivity to 3-BP [28]. It was also shown using the KBM7 human myeloid leukemia cell line, that MCT1 mediates the uptake of 3-BP and that deletion of the *MCT1* results in resistance to 3-BP [29]. Moreover, it was recently presented that higher expression of MCT1 in multiple myeloma cells correlated with increased uptake of 3-BP, when compared to the control cells [30]. The characteristics of transport of 3-BP into human erythrocytes was described very recently [31]. Our recent research on 3-BP using the yeast model showed that it enters the yeast cell mainly through the lactate/pyruvate-H+ permease Jen1p and that it is not a substrate for the multidrug resistance PDR efflux pumps [26]. We also showed that 3-BP is a potent antifungal agent with very selective toxicity toward the human pathogen *Cryptococcus neoformans* [32].

This is the first report showing multidirectional influence of 3-BP on the yeast *Saccharomyces cerevisiae*, a very useful model system for genomic screen of deletion mutants defective in the energetic metabolism.

## RESULTS

### General toxicity of 3-BP in the yeast *Saccharomyces cerevisiae* and its influence on the intracellular ATP levels

It was crucial to determine general toxicity of 3-BP in *Saccharomyces cerevisiae* strains and its influence on the intracellular ATP levels. The minimal synthetic medium (SD) was chosen for all of the experiments and different carbon sources were tested using the spot-test method in the wild-type W303-1A strain. These preliminary tests showed that glucose and other repressing sugars cause relative insensitivity to 3-BP (no visible effect at concentrations up to 3 mM in the wild-type), whereas nonfermentable and non-repressing carbon sources render wild-type yeast sensitive to 3-BP in the range of 1.5-2.5 mM, depending on a strain. These results suggested that the uptake mechanism of 3-BP could be glucose-repressible. Henceforth, most of the experiments were performed using sucrose as a sole carbon source in culture media, unless it is stated differently.

**Figure 1:**
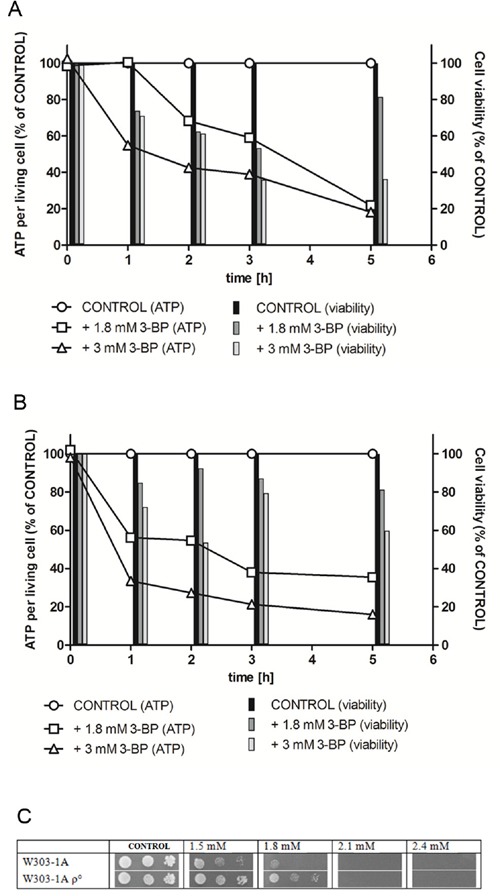
Intracellular ATP levels and viability in the parental wild type W303-1A A. and W303-1A rho0 mutant B. strains are decreased under 3-BP treatment The cells were incubated for 5 hours in minimal medium with sucrose with 1.8 mM 3-BP and 3 mM 3-BP. Bars represent viability of the cells at each time-point. The ATP levels are recalculated per living cells, taking the positive control (medium without 3-BP) as 100%. **C.** A spot-test showing 3-BP sensitivity in the wild-type W303-1A strain and the W303-1A rho^0^ respiratory mutant. Incubation time: 72 h.

Direct impact on cellular ATP levels and cell viability in liquid culture of yeast during the first 5 hours of exposition was determined. 3-BP concentrations of 1.8 mM (a sub-MIC value) and 3 mM (1.5-fold MIC) were chosen for the experiment. ATP level values shown are recalculated per living cell, to show the change in ATP level in living cells, neglecting the ATP fall caused by decreasing amount of cells being alive. The wild-type W303-1A (Figure 1A) and a respiratory deficient mutant (containing no mitochondrial DNA) W303-1A rho^0^ (Figure 1B) were chosen for the test.

In the wild-type W303-1A in the first hour of the experiment, concentration of 1.8 mM of 3-BP did not cause any effect on the intracellular ATP levels, however it decreased the strain viability to about 70%. 3 mM caused similar viability decrease, however simultaneous fall to under 60% in the level of ATP occurred. In the W303-1A rho^0^ respiratory mutant the effect on the ATP levels was significantly stronger (about 60% and 35% of the control, for 1.8 mM and 3 mM 3-BP, respectively), however showing weaker influence on viability. In the second and third hour of incubation further decrease in ATP levels was shown for both strains, however viability was more affected in the wild-type W303-1A. In the fifth hour of the experiment both concentrations of 3-BP dropped the ATP levels to about 20% of control in the wild-type strain. Also in the respiratory deficient mutant 3 mM of 3-BP caused decrease of ATP to about 20%, however 1.8 mM 3-BP only to about 35%. The viability of both strains when treated with 1.8 mM was about 80%. When 3 mM 3-BP was used the wild-type viability decreased to under 40%, whereas the rho^0^ maintained viability of 60%.

It can be concluded that the decrease of ATP levels is more rapid in the rho^0^ respiratory-deficient mutant, however, it is not as strong as in the wild-type in the long-term perspective. Generally, the rho^0^ remained more viable during the experiment, regardless of the 3-BP concentration used, showing that the presence of functional mitochondria causes increased overall sensitivity to 3-BP.

The differences in susceptibility of the wild-type W303-1A and the mitochondrial respiratory mutant W303-1A rho^0^ to 3-BP were also tested using the spot-test method (Figure 1C). The respiratory mutant exhibited increased resistance to 3-BP comparing to wild-type W303-1A, confirming the results of experiment determining the ATP levels and viability after 3-BP treatment.

### Toxicity of 3-BP in yeast mutants with disrupted glycolytic and TCA enzymes

Taking into account that 3-BP acts on the energetic metabolism of the cell, an experiment was carried out in order to check whether absence of specific glycolytic and respiratory enzymes may have influence on susceptibility to 3-BP. A set of 40 yeast strains from the EUROSCARF collection having deleted single genes encoding proteins engaged in glycolysis and TCA cycle were tested.

The summary of spot-tests showing the effect of a specific mutation on 3-BP susceptibility is shown in Table 1.

**Table 1:** The summary of spot-tests showing the influence of 3-BP on yeast mutants with disrupted glycolytic and respiratory enzymes

STRAIN	DISRUPTED ENZYME	PHENOTYPE	C+	1.2 mM 3-BP	1.5 mM 3-BP	1.8 mM 3-BP	2.1 mM 3-BP	2.4 mM 3-BP	2.7 mM 3-BP
BY4741	Wild-type		+++	+++	+++	+++	+++	+	−
Δhxk1	hexokinase-1	−	+++	+++	+++	+++	+++	+	−
Δhxk2	hexokinase-2	−	+++	+++	+++	+++	+++	+	−
Δglk1	glucokinase	R	+++	+++	+++	+++	+++	++	−
**Δpfk1**	**phosphofructokinase alpha**	**HR**	+++	+++	+++	+++	+++	+++	+
Δpfk2	phosphofructokinase beta	R	+++	+++	+++	+++	+++	++	0
Δfbp1	fructose-1,6-bisphosphatase	R	+++	+++	+++	+++	+++	+++	0
Δtdh1	3-P-glyceraldehyde dehydrogenase	−	+++	+++	+++	+++	+++	+	0
Δtdh2	3-P-glyceraldehyde dehydrogenase	−	+++	+++	+++	+++	+++	+	0
Δtdh3	3-P-glyceraldehyde dehydrogenase	−	+++	+++	+++	+++	+++	+	0
Δeno1	enolase-1	R	+++	+++	+++	+++	+++	+++	0
Δeno2	enolase-2	−	+++	+++	+++	+++	+++	+	0
Δpyk2	pyruvate kinase	R	+++	+++	+++	+++	+++	++	0
Δcit1	citrate synthase	−	+++	+++	+++	+++	+++	+	0
Δcit3	citrate synthase	R	+++	+++	+++	+++	+++	++	0
Δaco1	aconitase	−	+++	+++	+++	+++	+++	+	
Δaco2	aconitate hydratase	S	+++	+++	+++	+++	+++	0	0
Δidh1	NAD-dependent isocitrate dehydrogenase	S	+++	+++	+++	+++	0	0	0
Δidh2	NAD-dependent isocitrate dehydrogenase	S	+++	+++	+++	+++	+	0	0
**Δkgd1**	**alpha-ketoglutarate dehydrogenase**	**HS**	+++	+++	+++	0	0	0	0
Δkgd2	alpha-ketoglutarate dehydrogenase	−	+++	+++	+++	+++	+++	+	0
Δlpd1	lipoamide dehydrogenase (subunit of mitochondrial PDH complex)	S	+++	+++	+++	+++	+	0	0
Δsdh1	succinate dehydrogenase flavoprotein subunit	S	+++	+++	+++	+++	++	0	0
Δmdh1	mitochondrial malate dehydrogenase	S	+++	+++	+++	+++	++	0	0
Δpdx1	pyruvate dehydrogenase complex protein X	−	+++	+++	+++	+++	+++	+	0
**Δpyc1**	**pyruvate carboxylase**	**HS**	+++	++	0	0	0	0	0
Δpyc2	pyruvate carboxylase	−	+++	+++	+++	+++	+++	+	0
Δdld1	D-lactate dehydrogenase	−	+++	+++	+++	+++	+++	+	0
Δcyb2	L-lactate cytochrome-c oxidoreductase	−	+++	+++	+++	+++	+++	+	0
Δpdc1	pyruvate decarboxylase	R	+++	+++	+++	+++	+++	+++	0
Δadh1	alcohol dehydrogenase	R	+++	+++	+++	+++	+++	++	0
Δacs1	acetyl-coA synthetase	−	+++	+++	+++	+++	+++	+	0
Δald4	mitochondrial aldehyde dehydrogenase	−	+++	+++	+++	+++	+++	+	0
Δald6	cytosolic aldehyde dehydrogenase	−	+++	+++	+++	+++	+++	+	0
Δicl1	isocitrate lyase	R	+++	+++	+++	+++	+++	++	0
Δmls1	malate synthase-1	−	+++	+++	+++	+++	+++	+	0
Δmdh2	cytoplasmic malate dehydrogenase	−	+++	+++	+++	+++	+++	+	0
Δpck1	phosphoenolpyruvate carboxykinase	−	+++	+++	+++	+++	+++	+	0
**Δzwf1**	**glucose-6-phosphate dehydrogenase**	**HS**	+++	++	+	0	0	0	0
Δtkl1	transketolase	S	+++	+++	+++	+++	++	0	0
Δtal1	transaldolase	S	+++	+++	+++	+++	+++	0	0

The 3-BP sensitive/resistant phenotype of each strain is specified, comparing to the wild-type BY4741. Comparison of 3-BP sensitivity in the wild-type BY4741 strain and isogenic mutants with deleted *HXK1*, *HXK2*, *GLK1*, *PFK1*, *PFK2*, *FBP1*, *TDH1*, *TDH2, TDH3, ENO1, ENO2, PYK2, CIT1, CIT3, ACO1, ACO2, IDH1, IDH2, KG1, KGD2, LPD1, SDH1, MDH1, PDX1, PYC1, PYC2, DLD1, CYB2, PDC1, ADH1, ACS1, ALD4, ALD6, ICL1, MLS1, MDH2, PCK1, ZWF1, TKL1* and *TAL1* genes.

S – sensitive; HS – highly sensitive; R – resistant; HR – highly resistant; - – no change comparing to wild type; +++ – growth from dilutions 10^0^, 10^−1^, 10^−2^; ++ – growth from dilutions 10^0^, 10^−1^; + – growth from dilution 10^0^; 0 – no growth.

Deletion of genes encoding cytosolic enzymes caused highly resistant phenotype in the case of phosphofructokinase alpha (*PFK1*) and moderate resistance in the case of glucokinase (*GLK1*), phosphofructokinase beta (*PFK2*), pyruvate kinase (*PYK2*), pyruvate decarboxylase (*PDC1*), alcohol dehydrogenase (*ADH1*), fructose-1,6-bisphosphatase (*FBP1*), enolase-1 (*ENO1*) and isocitrate lyase (*ICL1*). Hypersensitivity to 3-BP was observed in glucose-6-phosphate dehydrogenase (*ZWF1)* and pyruvate carboxylase (*PYC1*) mutants and moderate sensitivity in transketolase (*TKL1*) and transaldolase (*TAL1*).

Deletion of genes encoding mitochondrial enzymes and subunits caused high sensitivity in the case of alpha-ketoglutarate dehydrogenase (*KGD1*) and sensitive phenotype in the case of isocitrate dehydrogenases (*IDH1*) and (*IDH2*), lipoamide dehydrogenase (*LPD1*), aconitate hydratase (*ACO2*), mitochondrial malate dehydrogenase (*MDH1*) and succinate dehydrogenase flavoprotein subunit (*SDH1*) mutants. Resistant phenotype was observed in the case of citrate synthase (*CIT3*).

**Figure 2:**
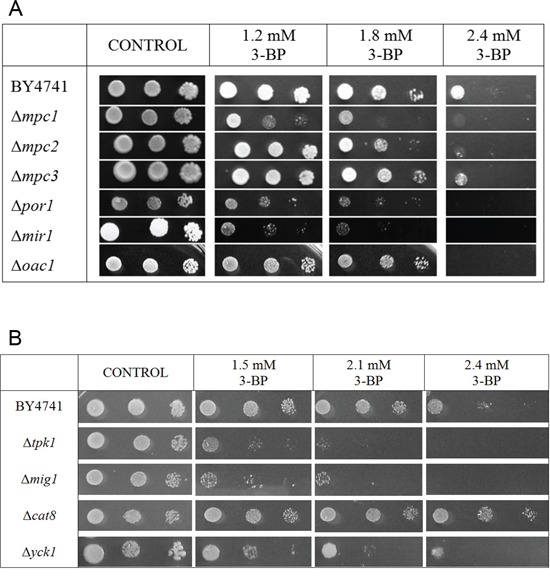
Influence of mutations in selected genes encoding factors involved in the regulation of the glucose-repression and mitochondrial pyruvate carrier as well as the mitochondrial porin, mitochondrial phosphate carrier and mitochondrial oxaloacetate carrier on sensitivity to 3-BP **A.** Deletion of each of the *POR1*, *MIR1* and *OAC1* genes resulted in increased sensitivity to 3-BP. The Δ*mpc1* mutant showed the strongest effect in increased of sensitivity to 3-BP, weaker effect was noted for Δ*mpc2*. Incubation time: 72 h. **B.** Among mutants which were deleted in genes encoding proteins involved in the glucose-repression/derepression system: *TPK1*, *MIG1*, *CAT8* and *YCK1* all tested strains showed sensitivity to 3-BP except Δ*cat8* which was resistance to 3-BP. Incubation time: 72 h.

As the metabolism and flux of pyruvate may be crucial to identify the mechanism of action of 3-BP, the influence of functionality of the Mitochondrial Pyruvate Carrier (Mpc) on susceptibility to 3-BP was checked. There are three genes in yeast taking part in encoding the mitochondrial pyruvate carrier, i.e., *MPC1*, *MPC2* and *MPC3*, with the first one being crucial for its function. The results of the performed spot-test are presented in Figure 2A. Comparing to the wild-type, the Δ*mpc1* mutant clearly showed increased sensitivity to 3-BP with just a minimal effect in the Δ*mpc2* strain and no visible effect in the Δ*mpc3* strain.

Three other mitochondrial membrane proteins, i.e., the mitochondrial porin Por1p (VDAC), mitochondrial phosphate carrier (Mir1p) and mitochondrial oxaloacetate carrier (Oac1p) were also tested (Figure 2A). Deletion of each of the *POR1*, *MIR1* and *OAC1* genes resulted in increased sensitivity to 3-BP.

The influence of deletion of genes encoding factors involved in the regulation of the glucose-repression system on 3-BP sensitivity was also tested. Δ*tpk1* strain, having inactive PKA (cAMP-dependent protein kinase) and both, Δ*mig1* and Δ*yck1* strain have defective glucose repression [33, 34]. Δ*cat8* cells cannot activate the gluconeogenic pathway and are unable to grow on unfermentable carbon sources, having faulty glucose derepression. Δ*tpk1*, Δ*mig1* and Δ*yck1* strains exhibited sensitivity to 3-BP, whereas Δ*cat8* showed resistance comparing to the wild type (Figure 2B).

### Overexpression of the Whi2p causes resistance to 3-BP in yeast cells

Using yeast multicopy genomic cDNA library (Lacroute) for FY1679-28C strain transformation, nine transformants hyper-resistant to 3-BP were isolated. The resistant phenotype of the selected transformants was confirmed using the spot-test method. Plasmid DNA from resistant transformants was isolated and sequenced. All of the isolated plasmids contained similar parts (lengths of 4 – 7 kb) of XV chromosome. The common part of these sequences was the *WHI2* gene.

It was shown that overexpression of Whi2p renders the cells elongated and 3-fold larger than the wild-type. Moreover, the cells are unable to complete cytokinesis and the budding pattern becomes polar instead of axial, resulting in filamentous growth [35]. It has been suggested that Whi2p connects Ras2p to the Ras-protein kinase A (PKA) signaling pathway, by influencing Ras2p localization. It was reported that lack of functional Whi2p results in actin aggregation, which may trigger apoptosis on the Ras-PKA pathway. It may also result in mitochondrial damage during the diauxic shift [36].

One of the pathways of the general stress response in yeast is through Msn2p and Msn4p transcription factors. They lead to the expression of many stress responsive genes through binding to the STRE (stress response elements). It was shown that STRE-mediated gene expression under stress conditions is strongly reduced in cells with deletion of the *WHI2* gene, comparing to the wild-type. Moreover Δ*whi2* cells reach the stationary phase several hours later than the wild-type. Furthermore, Δ*whi2* mutant is unable to accumulate storage glycogen [37]. Normally, when yeast cells are starved, they arrest in G1 phase of the cell cycle and change their metabolism to be able to survive the unfavorable conditions. Δ*whi2* mutant cells do not exhibit this response to starvation, they carry on dividing and the mutant cells in stationary phase become much smaller than in WT. They also often arrest randomly in the cell cycle [38].

It was also shown that Whi2p is required for the TOR-controlled induction of mitophagy of the dysfunctional mitochondria [39]. However, solely fragmentation of mitochondria is not enough to trigger its degradation. The pathway of mitophagy is independent of the mechanism of mitochondrial fission and it is suggested that Whi2p links the mitophagy machinery to the Ras-PKA signaling pathway [45].

To confirm that Whi2p is engaged in resistance to 3-BP, a mutant strain with *WHI2* gene deleted was tested.

**Figure 3:**
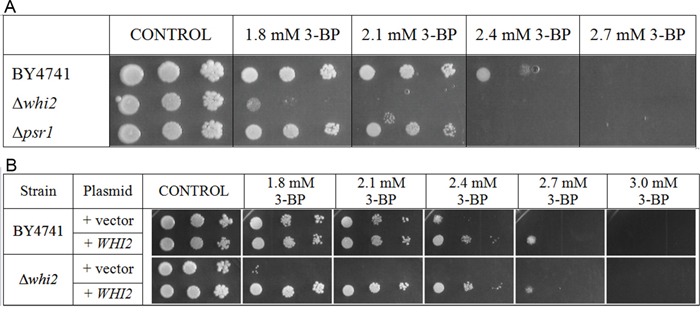
Δ*whi2* deletion mutant showed sensitivity to 3-BP in BY4741 background **A.** Comparison of 3-BP sensitivity in the wild-type BY4741 strain and isogenic mutants with *WHI2* and *PSR1* genes deleted. Deletion of *WHI2* gene strongly increased susceptibility to 3-BP, in contrast to **Δ***psr1* where observed sensitivity is weaker. Incubation time: 72h. **B.** The wild-type BY4741 strain and isogenic Δ*whi2* strain were transformed with a multicopy plasmid (pFl44L) containing *WHI2* gene, and as a control with an empty plasmid. Incubation time: 72h.

The Δ*whi2* strain in comparison to the wild-type shows strongly increased susceptibility to 3-BP (Figure 3A). However, growth of the Δ*psr1* mutant (which has deletion of the *PSR1* gene, encoding a binding partner of Whi2p) was only slightly weaker than of the wild-type.

To check whether expression of the *WHI2* gene from plasmid restores the resistance to 3-BP in the Δ*whi2* mutant, a spot-test using the Δ*whi2* and the wild-type strain with multicopy plasmid containing the *WHI2* gene and an empty pFL44L plasmid as a control was carried out. The presence of a multicopy plasmid with the *WHI2* gene increased resistance to 3-BP almost equally, both in the wild type BY4741 and the Δ*whi2* strain (Figure 3B).

It can be concluded that presence of Whi2p phosphatase is essential for resistance to 3-BP in yeast, as its overexpression increases resistance to whereas its deletion results in greater susceptibility to 3-BP.

### Deletion of both Whi2p binding partners, Psr1p and Psr2p, causes increased sensitivity to 3-BP

The plasma membrane phosphatases Psr1p and Psr2p were found (using the two-hybrid system) to interact directly with Whi2p [40]. The double Δ*psr1*Δ*psr2* mutant exhibits similar phenotype properties to the Δ*whi2* strain, i.e., reduced STRE-mediated gene expression, hyperphosphorylation of the Msn2p/Msn4p factors, higher sensitivity to stress (such as heat shock or sodium stress). Interaction of Psr1p, Msn2p and Whi2p was shown by immunoprecipitation experiments [43]. It is possible that Whi2p is a necessary adaptor between Msn2p and Psr1p which allows direct dephosphorylation of Msn2p by Psr1p phosphatase. These data suggest that Whi2p together with Psr1p/Psr2p are needed for activation of the general stress response by interacting with Msn2p [43]. However, Whi2p and Psr1p/Psr2p are just three of many other activators of Msn2p/Msn4p pathway (e.g., Mck1p, Snf1p, Hog1p kinases) [41].

**Figure 4:**
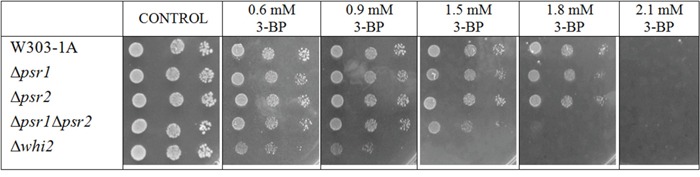
In W303 background Δ*whi2* as well as Δ*psr1*Δ*psr2* mutants showed sensitivity to 3-BP, whereas Δ*psr1* did not Comparison of 3-BP sensitivity between the wild-type W303-1A and isogenic Δ*whi2*, Δ*psr1*, Δ*psr2* and Δ*psr1*Δ*psr2* strains. Incubation time: 72 h.

To check whether Whi2p-mediated resistance occurs on the known pathway due to its binding with Psr1p/Psr2p phosphatases, single Δ*psr1* and Δ*psr2* mutants and a double mutant Δ*psr1*Δ*psr2* in the W303-1A background were constructed and tested (Figure 4). None of the single Δ*psr1* and Δ*psr2* mutants showed different sensitivity to 3-BP than the wild-type. This is different than in the BY4741 background, where the single Δ*psr1* showed slightly increased sensitivity. However, in the W303-1A background, double Δ*psr1*Δ*psr2* mutant showed increased susceptibility to 3-BP comparing to wild-type and the single-mutants. It should be noticed, this effect was not as strong as in Δ*whi2* mutant.

### Whi2-mediated resistance to 3-BP does not occur through interaction with the HOG stress response pathway

The following proteins: Whi2p/Psr1p, Msn2p, Msn4p and Hog1p are essential for the initiation of general stress response pathway in yeast [42]. Under stress condition Whi2p/Psr1p phosphatase complex probably activates Msn2p and Msn4p, which may activate the Hog1p kinase.

**Figure 5:**
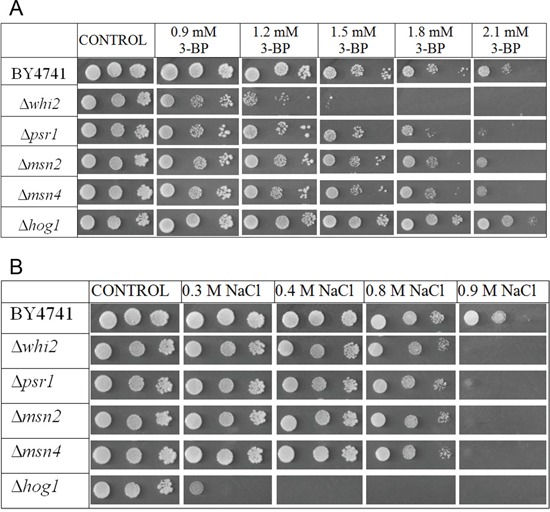
Whi2p-mediated resistance to 3-BP probably does not occur via the HOG stress response pathway **A.** Comparison of 3-BP sensitivity in the wild-type BY4741 strain and isogenic mutants with *WHI2*, *PSR1*, *MSN2*, *MSN4* and *HOG1* genes deleted. **B.** Strainscontrol on medium with sodium chloride. Incubation time: 72 h.

**Figure 6:**
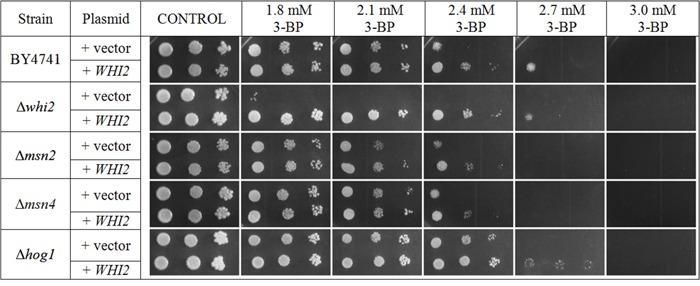
Transformation with multicopy plasmid containing the *WHI2* gene improves resistance to similar levels of 3-BP in every strain tested (Δ*whi2*, Δ*msn2*, Δ*msn4*, WT) Growth of BY4741, Δ*whi2*, Δ*msn2*, Δ*msn4* and Δ*hog1* strains carrying multicopy plasmid (pFL44L) containing *WHI2* gene or an empty plasmid as a control. Incubation time: 72 h.

Results in Figure 5A show that Whi2p-mediated resistance phenotype in yeast is probably independent of general stress response pathway. Deletions of genes encoding Msn2p and Msn4p do not seem to have any negative effects on yeast grown on 3-BP. Transformation with multicopy plasmid containing the *WHI2* gene improves resistance to similar levels in every strain tested (Δ*whi2*, Δ*msn2*, Δ*msn4*, WT) which also suggests that resistance to 3-BP conferred by Whi2p is not due to upregulation of the HOG pathway (Figure 6). What is more, strain with deleted *HOG1* gene (in BY4741 background) shows slightly increased resistance to 3-BP. As the HOG pathway is responsible for resistance to osmotic stress, growth characteristics were compared to growth on medium with sodium chloride as a control (Figure 5B). These results show that Whi2p-mediated resistance to 3-BP probably does not occur via the HOG stress response pathway. Increased resistance may occur either because of direct Whi2p phosphatase activity or due to existence of other unidentified signaling pathway leading to other effectors than Hog1p.

### Whi2 and Jen1 influences on sensitivity to 3-BP seem to be independent

**Figure 7:**
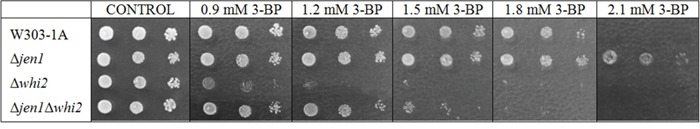
Function of Whi2 and Jen1 is independent in response to 3-BP Comparison of 3-BP sensitivity in the wild-type W303-1A and isogenic Δ*whi2*, *Δjen1* and a double Δ*jen1*Δ*whi2* mutant. Incubation time: 96 h.

As the Whi2p has a pleiotropic activity, it was checked if its influence on 3-BP sensitivity is connected with its possible regulatory effect on expression and/or activity of the Jen1p permease, which was shown to transport 3-BP in yeast [26, 43]. To check whether the resistance caused by deletion of *JEN1* is connected with the resistance caused by the overexpression of *WHI2*, a single Δ*whi2* mutant and a double mutant Δ*jen1*Δ*whi2* were constructed in the W303-1A background (Figure 7).

Both, Δ*whi2* and double Δ*jen1*Δ*whi2* strains exhibited increased sensitivity to 3-BP comparing to the wild-type and a single Δ*jen1* mutant. However the Δ*jen1*Δ*whi2* strain was proportionally more resistant to 3-BP comparing to the Δ*whi2* strain, thus suggesting that the effect of Whi2p is probably independent of the effect of Jen1p.

## DISCUSSION

3-BP, a small-molecule alkylating agent has been shown to be a potent and specific anticancer drug. 3-BP differs in the mode of action from the currently available chemotherapeutics, as it directly targets the energy metabolism of a cancer cell. Inhibition of both glycolysis and mitochondrial oxidative phosphorylation leads to rapid ATP depletion and apoptotic or necrotic cell death. Moreover 3-BP exhibits little or no effect on normal healthy cells. In this study the characteristics of 3-BP toxicity in the yeast model are described, with the emphasis on its influence on energetic metabolism of the cell and chosen regulatory pathways.

The results of the experiment showing direct influence of 3-BP on intracellular ATP levels in yeast, suggest that the mitochondrial respiration enzymatic machinery may be more sensitive to 3-BP than glycolysis, still of course both pathways being inhibited, as it was shown to happen in human cancer cells [44, 30]. Despite the fact that in the first hour of incubation ATP decrease is stronger in rho^0^ respiratory deficient mutant, it retains higher ATP levels at the end of the incubation and shows higher viability than the wild-type strain. The ATP depletion in the first hour is probably stronger in rho^0^ cells because glycolysis is its main source of energy and in the wild-type it takes time to affect the mitochondria and deplete the respiratory substrates and the TCA intermediates. This can be also due to the fact that loss of the mitochondrial genome in rho^0^ mutant triggers global reprogramming of gene expression called the retrograde response, which is a set of signaling pathways leading from the mitochondria to the nucleus [45]. It triggers expression of the nuclear *CIT2* gene, encoding citrate synthase-2, to ensure proper levels of α-ketoglutarate, which is a crucial precursor for the amino acids biosynthesis [46]. It was also shown that rho^0^ cells dramatically induce expression of ABC transporter-encoding genes like *PDR5*, as well as exhibit elevated transcription of loci encoding enzymes involved in sphingolipid biosynthesis [47]. This is however not in this case, as it was shown that 3-BP is not a substrate for the yeast PDR system [26]. The retrograde regulation, which has been shown to be controlled by the Rtg1p, Rtg2p and Rtg3p factors, results in increased expression of genes involved in anaplerotic pathways, transport of small molecules, as well as upregulated peroxisomal activities and stress responses. It increases over 10-fold expression of *CIT2* gene (encoding a glyoxylate cycle citrate synthase), *DLD3* (encoding a cytosolic D-lactate dehydrogenase) and *PDH1* (encoding protein involved in propionate metabolism) [48]. In cells with dysfunctional mitochondria expression of genes encoding enzymes of the TCA cycle that lead to the production of α-ketoglutarate (i.e., *CIT1*, *ACO1*, *IDH1* and *IDH2*) is dependent on the *RTG* genes, unlike cells with normal mitochondrial function, where they are regulated by *HAP2,3,4,5* transcription complex [49]. Independent regulation of these genes, as well as upregulation of the anaplerotic pathways may be responsible for increased resistance to 3-BP of the rho^0^ cells. The 3-BP-resistant phenotype of rho^0^ mutants was also shown in *Candida glabrata* (unpublished data). The expression of pyruvate carboxylase (*PYC1*) and acetyl-CoA synthase (*ACS1*) is also upregulated in rho^0^ cells. Moreover, flux of metabolites from β-oxidation of fatty acids and glyoxylate cycle are upregulated [54]. Pyc1p supplies oxaloacetate for gluconeogenesis and to replenish tricarboxylic acid cycle intermediates [50]. Overexpression of *PYC1* could be a probable determinant of 3-BP-resistance in the rho^0^ cells, as its deletion causes sensitivity to 3-BP.

Concerning the first step of glycolysis in yeast, hexokinase-2 (Hxk2p) is the predominant isoform when glucose is abundant, however upon glucose depletion its expression is repressed and hexokinase-1 (Hxk1p) and glucokinase (Glk1p) become active. Hxk1p and Glk1p function redundantly, their expression is upregulated in the absence of the other one [51]. Though not strong, resistance of Δ*glk1* strain to 3-BP may suggest that Glk1p could be more susceptible to inhibition by 3-BP than Hxk1p. In human cells it is proposed that hexokinase-2 is the isoform sensitive to 3-BP [20].

Glucose-6-phosphate is a substrate for further glycolytic processing, however, it may be entering the pentose phosphate pathway (PPP) instead. Deletion of the *ZWF1* gene encoding 6-phosphoglucose dehydrogenase, the enzyme catalyzing the first and rate-limiting step of the pentose phosphate pathway, resulted in increased sensitivity to 3-BP. Similar effect was visible in the Δ*tal1* mutant, lacking the enzyme transaldolase, which creates a reversible link between two the PPP and glycolysis [52]. The 3-BP-sensitive phenotype which is observed when the pentose phosphate pathway is disrupted may be explained by the fact, that PPP is a crucial source of NADPH, which acts as a cellular reducing power. NADPH provides the reducing potential for most of the antioxidant enzymes, such as glutathione and thioredoxin systems. As pentose phosphate pathway is directly connected to the glycolytic pathway, any change in activity of glycolytic enzymes may influence the flux of PPP, resulting in change of the amounts of NADPH produced [53]. It was previously shown that disruption of the *ZWF1* gene resulted in decreased resistance to oxidants, confirming the role of PPP in the oxidant tolerance mechanism [54]. Gorsich et al. showed that PPP mutants were inefficient at reducing furfural resulting in higher sensitivity to this inhibitor, as well as to 5-hydroxymethylfurfural [55]. Moreover, previous studies showed correlation between resistance to sorbic acid and activity of several PPP enzymes [56].

The lack of a phosphofructokinase (*PFK*) probably forces the upregulation of the PPP, resulting in higher levels of NADH, which leads to enhanced glutathione reduction and increased 3-BP resistance. It is also known that strain with deleted *PFK1* gene, encoding the catalytic subunit of phosphofructokinase-1 show no Pasteur effect [57].

Moreover, induction of gluconeogenesis leads to a higher production of glucose-6-phosphate, the key substrate for the pentose phosphate pathway [58]. This would be confirmed by the 3-BP-sensitive phenotype of the Δ*pyc1* (pyruvate carboxylase-1), as this mutation blocks formation of oxaloacetate from pyruvate, which is a crucial step of gluconeogenesis. In the case of Δ*pyc1* the effect may be also due to the decreased anaplerosis of the TCA cycle, caused by low levels of oxaloactetate, which hypothesis is supported by 3-BP-sensitivity of the Δ*oac1* mutant lacking the mitochondrial oxaloacetate carrier [59].

On the other hand, lack of other gluconeogenic enzyme, namely fructose-1,6-bisphosphatase (Fbp1p), resulted in resistance to 3-bromopyruavte. Expression profiles showed that genes induced by the diauxic shift are induced by oxidative stress, DNA damage and other stress conditions [63]. It was shown that fructose-1,6-bisphosphatase is also involved in ROS production in chronologically aged cells and after MMS treatment. Absence of *FBP1* resulted in better survival of cells treated with MMS, improved surviving of aged cells and delayed the induction of ROS production [63], which is consistent with the 3-BP-resistant phenotype.

Disruption of pyruvate decarboxylase (*PDC*) and aldehyde dehydrogenease (*ADH*) causes the pyruvate to enter the TCA cycle or gluconeogenesis rather than the pathway of ethanol production, which agrees with the fact that active glucoeneogenesis seems to diminish the toxicity of 3-BP. Deletion of gene enoding pyruvate kinase (*PYK2*) results in increased resistance to 3-BP, possibly also beacuse phospho-enolpyruvate is forced into the gluconeogenic pathway.

Deletion of genes encoding TCA enzymes, i.e., *ACO2*, *IDH1*, *IDH2*, *KGD1*, *LPD1*, *SDH1* and *MDH1* generally resulted in increased sensitivity to 3-BP also suggesting that preservation of the functional TCA cycle flux is important for preventing the 3-BP-induced toxicity. The Δ*kgd1* strain lacking the activity of mitochondrial alpha-ketoglutarate dehydrogenase is a respiratory deficient mutant and it does not utilize glycerol, however it has a functional respiratory chain and synthesis of other mitochondrial enzymes is not affected. In this mutant expression levels of aconitase, isocitrate dehydrogenase, succinate dehydrogenase and malate dehydrogenase as well as dihydrolipoyl dehydrogenase were not different from the wild-type [60]. It is possible that deletion of *KGD1* as well as other genes encoding enzymes of the TCA cycle forward of the isocitrate dehydrogenase resulted in sensitivity to 3-BP due to activation of the glyoxylate cycle. This would be supported by the fact that deletion of the *ICL1* gene, encoding isocitrate lyase (the crucial enzyme in the glyoxylate cycle), resulted in resistance to 3-BP.

Deletion of *CIT3* gene, encoding a mitochondrial citrate synthase, has no effect on the citrate/methylcitrate synthase activity, as the other isoforms are active. However, Cit3p is essential for growth on propionate as a carbon and energy source. It was shown that Δ*cit3* exhibits unusual pyruvate metabolism with dramatic accumulation of acetate and isobutanol, which is probably caused by excessive mitochondrial propionyl-CoA, resulting in inhibition of the PDH E2 subunit [61]. The Δ*cit3* strain exhibited resistance to 3-BP which may be explained by the fact, that accumulation of acetate occurring in this strain may lead to the downregulation of pyruvate decarboxylation and increased which directs pyruvate rather to mitochondria or to the gluconeogenic pathway.

It was shown that deletion of *MPC* genes, encoding mitochondrial pyruvate carrier, results in impaired pyruvate metabolism, accumulation of upstream metabolites and depletion of TCA cycle intermediates. Δ*mpc* mutants exhibited increased cytoplasmic pyruvate concentrations, depleted malate and acetyl-CoA, suggesting that they are unable to properly convert pyruvate to mitochondrial acetyl-CoA, which results in nonfunctional TCA cycle and impaired ATP production. Uptake assay on isolated mitochondria showed almost no uptake of ^14^C-pyruvate in the Δ*mpc1* strain [62]. The sensitivity to 3-BP of the Δ*mpc1* and Δ*lpd1*, together with resistance of the Δ*pdc1*, may suggest that increased activity of the PDH-bypass pathway possibly increases 3-BP toxicity.

Δ*por1* and Δ*mir1* mutants, lacking mitochondrial porin and phosphate carrier (being the parts of the ATP-synthasome), show sensitivity to 3-BP, possibly due to partially disrupted respiration, as well as an imbalance of NAD/NADH, resulting in insufficient reducing buffer.

**Figure 8:**
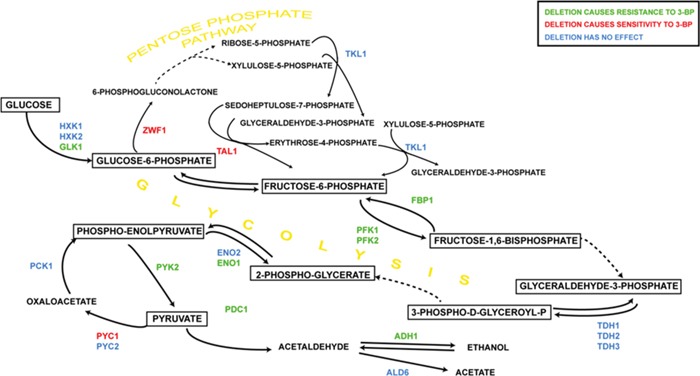
The influence of 3-BP on single yeast mutants having deleted genes encoding glycolytic and pentose phosphate pathway enzymes Deletion of genes marked in: green resulted in increased resistance to 3-BP; red – increased sensitivity; blue – no changes comparing to wild-type.

Abbreviations: ADH1, alcohol dehydrogenase; ALD6, cytosolic aldehyde dehydrogenase; ENO1, enolase-1; ENO2, enolase-2; FBP1, fructose-1,6-bisphosphatase; GLK1, glucokinase; HXK1, hexokinase-1; HXK2, hexokinase-2; PCK1, phosphoenolpyruvate carboxykinase; PDC1, pyruvate decarboxylase-1; PFK1, phosphofructokinase alpha subunit; PFK2, phosphofructokinase beta subunit; PYC1, pyruvate carboxylase-1; PYC2, pyruvate carboxylase-2; PYK2, pyruvate kinase; TAL1, transaldolase; TDH1, 3-P-glyceraldehyde dehydrogenase-1; TDH2, 3-P-glyceraldehyde dehydrogenase-2; TDH3, 3-P-glyceraldehyde dehydrogenase-3; TKL1, transketolase; ZWF1, glucose-6-phosphate dehydrogenase.

**Figure 9:**
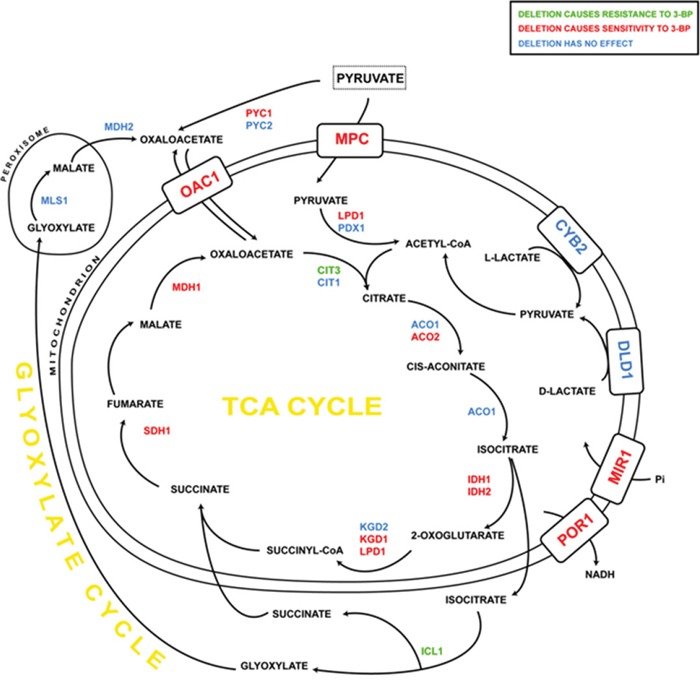
The influence of 3-BP on single yeast mutants having deleted genes encoding TCA cycle and glyoxylate cycle enzymes, as well as mitochondrial carriers Deletion of genes marked in: green resulted in increased resistance to 3-BP; red – increased sensitivity; blue – no changes comparing to wild-type.

Abbreviations: ACO1, aconitase; ACO2, aconitate hydratase; CIT1, citrate synthase; CIT3, citrate synthase; CYB2, L-lactate cytochrome-c oxidoreductase; DLD1, D-lactate dehydrogenase; ICL1, isocitrate lyase; IDH1, NAD-dependent isocitrate dehydrogenase; IDH2, NAD-dependent isocitrate dehydrogenase; KGD1, alpha-ketoglutarate dehydrogenase; KGD2, alpha-ketoglutarate dehydrogenase; LPD1, dihydrolipoamide dehydrogenase; MDH1, mitochondrial malate dehydrogenase; MDH2, cytoplasmic malate dehydrogenase; MIR1, mitochondrial phosphate carrier; MLS1, malate synthase; MPC, mitochondrial pyruvate carrier; OAC1, oxaloacetate transporter; PDX1, pyruvate dehydrogenase complex protein X; POR1, mitochondrial porin (voltage-dependent anion channel); PYC1, pyruvate carboxylase-1; PYC2, pyruvate carboxylase-2; SDH1, succinate dehydrogenase.

It can be concluded that when grown on medium with 3-BP, single mutants concerning the TCA cycle enzymes exhibit different phenotypes than rho^0^ strains, possibly due to the retrograde response activated in the latter. Generally, single deletions of genes encoding the TCA cycle enzymes and mitochondrial carriers resulted in increased sensitivity to 3-BP, whereas mutations in the glycolytic pathway resulted in increased resistance (Figures 8 and 9). On the other hand disruption of the pentose phosphate pathway resulted in increased sensitivity. It is probable that most of the effects of these mutations come down to the upregulation of gluconeogenesis and pentose phosphate pathway which is the main source of NADPH which provides the reducing potential for most of the antioxidant agents, such as glutathione, which has great influence on 3-BP susceptibility [26]. Moreover, also the TCA cycle replenishes the pool of NADPH through mitochondrial transhydrogenase, thus having an impact on cellular glutathione levels [63].

It cannot be also excluded that 3-bomopyruvate could be recognized as a mimicking substrate for one or more enzymes engaged in metabolism of pyruvate. It has been shown in vitro that lactate dehydrogenase (LDH) may use 3-BP as a substrate, however with over 100-fold lower affinity than pyruvate (unpublished data).

Yeast cells grown on medium with high concentration of glucose during the logarithmic phase produce energy mainly by fermentation of glucose, whereas respiration and stress response mechanisms are repressed (the Crabtree effect). When glucose is exhausted the diauxic shift occurs, resulting in changes in expression levels of many proteins as well as the upregulation of stress response pathways [42]. Cells switch to metabolism of unfermentable carbon sources, gluconeogenic, TCA cycle, and glyoxylate shunt genes are induced [64]. The expression of *PCK1* (phosphoenolpyruvate carboxykinase), *FBP1* (fructose-1,6-bisphosphatase), *MDH2* (malate dehydrogenase), as well as *ICL1* (isocitrate lyase) and *MLS1* (malate synthase) are upregulated during glucose depletion [65]. In yeast the Ras-cAMP-PKA signaling pathway is essential for the diauxic shift, it also regulates mitochondrial function and ROS production. PKA (cAMP-dependent protein kinase) is inactive during unfermenting growth and is activated when glucose becomes abundant and lack of PKA results in defective glucose repression [66]. The Δ*tpk1* strain, lacking the subunit of PKA, shows increased sensitivity to 3-BP, which may be due to the defect in glucose repression mechanism. In yeast cells grown in the absence of glucose, on media with alternative sugars, such as sucrose, galactose, and maltose, Mig1p repressor activity is inhibited. In the presence of glucose Mig1p factor localizes to the nucleus repressing expression of target genes such as *SUC2* encoding invertase [42]. Cat8p is one of the factors required for activation of gluconeogenesis during growth on unfermetable carbon sources, therefore Δ*cat8* cells are unable to grow on unfermentable carbon sources [69]. *CAT8* transcription is inhibited by Mig1p and activated by the Hap2/3/4/5p complex. Δ*cat8* mutant, exhibited increased resistance to 3-BP, whereas Δ*mig1* strain, having defective glucose repression, showed increased sensitivity to 3-BP when grown on sucrose. Similarly, the Δ*yck1* strain, exhibiting faulty respiration repression by glucose [39, 40], showed increased sensitivity to 3-BP. These results confirm that glucose repression reduces sensitivity to 3-BP, which effect is clearly visible in wild-type strains grown on glucose.

PKA is also engaged in activation of metabolic flux through glycolysis. PKA activates phosphofructokinase-2 (*PFK2*) [67], and indirectly also pyruvate kinase (*PYK2*) through increased production of fructose-2,6 bisphosphate which is an allosteric activator of pyruvate kinase [68]. PKA also phosphorylates the key gluconeogenic enzyme fructose-1,6-bisphosphatase (*FBP1*) leading to its degradation [42]. Sensitivity of Δ*tpk1* cells to 3-BP may be connected to its interaction with phosphofructokinase-2 and pyruvate kinase. Lack of PKA activity causes decreased activity if these enzymes giving phenotypic effect similar to deletion of the corresponding genes. This however seems not to be the case, as both Δ*pfk2* and Δ*pyk2* strains exhibited increased resistance to 3-BP.

The most probable pathway of PKA influence on 3-BP susceptibility is connected with Whi2p phosphatase and the mechanism of mitophagy. Cells without functional Whi2p fail to enter the stationary phase. Whi2p was suggested to be engaged in the cell cycle control, as Δ*whi2* cells fail to enter G1 and arrest randomly within the cell cycle in stationary phase [45]. These cells also fail to sense nutritional deprivation, leading to continuous proliferation. Sensitivity to 3-BP of the Δ*whi2* which was visible even when grown on medium with glucose, may be due to faulty glucose-repression system in this mutant. Whi2p together with Psr1p and Psr2p mediate the general response to environmental stress through the Msn2p/Msn4p factors [43]. It can, however, be excluded that *WHI2*-related sensitivity to 3-BP is a consequence of disrupted general stress response pathway, as the Δ*msn2*/Δ*msn4* and Δ*hog1* strains do not exhibit sensitivity to 3-BP. Deletion of *PSR1* and *PSR2* genes encoding Whi2p binding partners resulted in increased sensitivity suggesting, that Psr1p/Psr2p are required for the Wh2p-mediated response to 3-BP.

Moreover, it was shown that lack of Whi2p leads to actin aggregation, which is thought to induce apoptosis, probably occurring on the Ras-cAMP-PKA pathway [42]. Δ*whi2* cells exhibit nuclear fragmentation and loss of mitochondrial DNA during diauxic shift. The Δ*whi2* strain was shown to be sensitive to 2 mM H_2_O_2_, unlike the wild-type. Mitochondria in Δ*whi2* cells were reported to have reduced membrane potential, exhibit fragmentation and production of high levels of ROS during the diauxic shift [42]. It has been recently explained to be a consequence of defective mitophagy on the Ras-cAMP-PKA pathway in Δ*whi2* cells, resulting in reduced elimination of damaged mitochondria [45]. This seems to be the most probable reason for 3-BP-sensitivity of Δ*whi2* and Δ*tpk1* (PKA) strains.

As the turnover of Jen1p was shown to be regulated through phosphorylation by the Yck1p kinase [69], it was possible that the phosphatase activity of Whi2p could influence the activity of Jen1p. This however is not the case, as the double Δ*jen1*Δ*whi2* mutant shows similar increase in resistance to 3-BP comparing to the single Δ*whi2*, as the Δ*jen1* mutant to wild-type.

Concluding, this study shows the multidirectional influence of 3-BP on metabolism in yeast. The lack of glucose-repression mechanism activity is crucial for 3-BP toxicity, which is mainly due to ATP depletion. Glucose present in the culturing medium, as well as genetic disruption of the glucose-repression system decreases toxic activity of 3-BP. Generally single deletions of genes encoding the TCA cycle enzymes and mitochondrial carriers cause sensitivity to 3-BP. Disruption of the pentose phosphate pathway results in increased sensitivity to 3-BP, probably due to the fact that PPP is a main source of NADPH which provides the reducing potential for most of the antioxidant agents, including glutathione. Most of the effects on 3-BP-sensitivity of the single disruptions in glycolytic and TCA pathways may probably be explained by down- or upregulation of the gluconeogenesis and pentose phosphate pathway and its influence on the reducing buffer of the cell. Presence of yeast Whi2p phosphatase is essential for resistance phenotype to 3-BP, probably because of its interaction with glucose-repression mechanism and the mitophagy of damaged mitochondria through the Ras-cAMP-PKA pathway. It is noteworthy that Whi2-mediated resistance to 3-BP does not occur through the HOG stress response pathway. Moreover, the effect of 3-BP resistance caused by disruption of the Jen1p transporter disruption is independent of the sensitivity effect caused by the deletion of *WHI2* gene. The results obtained in this study may help elucidate the mechanisms of activity of 3-BP on glycolytic and TCA enzymes as well as its influence on yeast mitochondria and glucose repression system. However, further research is needed concerning the influence of faulty mitophagy on 3-BP toxicity. These findings may help to better understand the toxic activity of 3-BP and improve its effectiveness as an anticancer and antifungal drug.

## MATERIALS AND METHODS

### Yeast and bacterial strains used in this study

The yeast *Saccharomyces cerevisiae* haploid strains used in this study are listed in Table 4.

**Table 2:** Plasmids used in this study

Plasmid	Description	Reference
pFL44L	YE-type high copy number shuttle vector, *URA3*, Amp^R^	Bonneaud et al. [72]
pFA6a-kanMX6	Vector containing *kanMX6* cassette for gene deletion, Amp^R^	Wach et al. [77]
pFA6a-TRP1	Vector containing *TRP1* cassette for gene deletion, Amp^R^	Longtine et al. [73]
BPR1	pFL44L containing ∼4000bp fragment of XV yeast chromosome containing the complete *WHI2* gene and a fragment of *CUE5* gene	This study

**Table 3:** Primers used in this study

Primer	Sequence 5′ – 3′
WHI2F1	ATGGACGATATAATCACGCAAGTTTCTCCAGATAATGCAGCGGATCCCCGGGTTAATTAA
WHI2R1	TCACTGCACCCCAATAACGCTCAACTCTAAAGTCCAAACTGAATTCGAGCTCGTTTAAAC
WHI2PR	GGCATAGTGATAGAGTGTGA
WHI2INT1	TCCTGCTCATTGTTGCTCGA
PSR1F1	ATGGGTTTCATATCGTCAATACTGTGCTGCTCTTCCGAGACGGATCCCCGGGTTAATTAA
PSR1R1	TTATATTGTTACATCCAAAATTTTGCCCACATCCAGTGAGGAATTCGAGCTCGTTTAAAC
PSR1PR	AGGACGTATCACGTGACACAAAC
PSR1INT1	GAAGTATCTTTCACAGCATC
PSR2F1	ATGGGATTTATAGCAAATATACTGTGCTGTTCTTCAGATACGGATCCCCGGGTTAATTAA
PSR2R1	CTATATCGTCACATCCAACACGCTCCCCACATCCAACACGGAATTCGAGCTCGTTTAAAC
PSR2PR	TATCTTTTTCGCAGCGCGTA
PSR2INT1	AGACAAAAGGATCTCCGGTGT
PKAN	GATTGCCCGACATTATCGCGAG
PTRP1	GTTGCAGTCTTTTGGAAATAC
PTRP2	CTCCAAGCTGCCTTTGTGTG
M13FWD	GTAAAACGACGGCCAGT
M13REV	CAGGAAACAGCTATGAC

**Table 4:** Yeast strains used in this study

Strain	Genotype	Reference
**W303-1A**	**MAT a, *ade2-1, leu2-112, his3-11,15, trp1-1, ura3-1***	Rothstein & Thomas [70]
W303-1A, rho^0^	**W303-1A**, *rho^0^*	This study
Δ*jen1* (BLC203)	**W303-1A**, Δ*jen1::HIS3*	Casal et al. [43]
Δ*whi2*	**W303-1A**, Δ*whi2::KanMX6*	This study
Δ*jen1*Δ*whi2*	**W303-1A**, Δ*jen1::HIS3*, Δ*whi2::KanMX6*	This study
Δ*psr1*	**W303-1A**, Δ*psr1::KanMX6*	This study
Δ*psr2*	**W303-1A**, Δ*psr2::KanMX6*	This study
Δ*psr1*Δ*psr2*	**W303-1A**, Δ*psr1::KanMX6*, Δ*psr2::TRP1*	This study
**BY4741**	**MAT a, Δ*his3*; Δ*leu2*; Δ*met15*; Δ*ura3***	Euroscarf
Δ*whi2*	**BY4741**, Δ*whi2*	Euroscarf
Δ*psr1*	**BY4741**, Δ*psr1*	Euroscarf
Δ*msn2*	**BY4741**, Δ*msn2*	Euroscarf
Δ*msn4*	**BY4741**, Δ*msn4*	Euroscarf
Δ*hog1*	**BY4741**, Δ*hog1*	Euroscarf
Δ*hxk1*	**BY4741**, Δ*hxk1*	Euroscarf
Δ*hxk2*	**BY4741**, Δ*hxk2*	Euroscarf
Δ*glk1*	**BY4741**, Δ*glk1*	Euroscarf
Δ*pfk2*	**BY4741**, Δ*pfk2*	Euroscarf
Δ*aco2*	**BY4741**, Δ*aco2*	Euroscarf
Δ*tdh1*	**BY4741**, Δ*tdh1*	Euroscarf
Δ*pyk2*	**BY4741**, Δ*pyk2*	Euroscarf
Δ*kgd1*	**BY4741**, Δ*kgd1*	Euroscarf
Δ*idh2*	**BY4741**, Δ*idh2*	Euroscarf
Δ*cit3*	**BY4741**, Δ*cit3*	Euroscarf
Δ*pyc2*	**BY4741**, Δ*pyc2*	Euroscarf
Δ*kgd2*	**BY4741**, Δ*kgd2*	Euroscarf
Δ*pyc1*	**BY4741**, Δ*pyc1*	Euroscarf
Δ*tdh3*	**BY4741**, Δ*tdh3*	Euroscarf
Δ*aco1*	**BY4741**, Δ*aco1*	Euroscarf
Δ*idh1*	**BY4741**, Δ*idh1*	Euroscarf
Δ*cit1*	**BY4741**, Δ*cit1*	Euroscarf
Δ*lpd1*	**BY4741**, Δ*lpd1*	Euroscarf
Δ*pfk1*	**BY4741**, Δ*pfk1*	Euroscarf
Δ*tdh2*	**BY4741**, Δ*tdh2*	Euroscarf
Δ*aco2*	**BY4741**, Δ*aco2*	Euroscarf
Δ*eno1*	**BY4741**, Δ*eno1*	Euroscarf
Δ*eno2*	**BY4741**, Δ*eno2*	Euroscarf
Δ*dld1*	**BY4741**, Δ*dld2*	Euroscarf
Δ*cyb2*	**BY4741**, Δ*cyb2*	Euroscarf
Δ*pdc1*	**BY4741**, Δ*pdc1*	Euroscarf
Δ*pdx1*	**BY4741**, Δ*pdx1*	Euroscarf
Δ*adh1*	**BY4741**, Δ*adh1*	Euroscarf
Δ*acs1*	**BY4741**, Δ*acs1*	Euroscarf
Δ*ald4*	**BY4741**, Δ*ald4*	Euroscarf
Δ*ald6*	**BY4741**, Δ*ald6*	Euroscarf
Δ*mpc1*	**BY4741**, Δ*mpc1*	Euroscarf
Δ*mpc2*	**BY4741**, Δ*mpc2*	Euroscarf
Δ*mpc3*	**BY4741**, Δ*mpc3*	Euroscarf
Δ*por1*	**BY4741**, Δ*por1*	Euroscarf
Δ*mir1*	**BY4741**, Δ*mir1*	Euroscarf
Δ*oac1*	**BY4741**, Δ*oac1*	Euroscarf
Δ*icl1*	**BY4741**, Δ*icl1*	Euroscarf
Δ*sdh1*	**BY4741**, Δ*sdh1*	Euroscarf
Δ*zwf1*	**BY4741**, Δ*zwf1*	Euroscarf
Δ*mls1*	**BY4741**, Δ*mls1*	Euroscarf
Δ*mdh1*	**BY4741**, Δ*mdh1*	Euroscarf
Δ*mdh2*	**BY4741**, Δ*mdh2*	Euroscarf
Δ*tal1*	**BY4741**, Δ*tal1*	Euroscarf
Δ*fbp1*	**BY4741**, Δ*fbp1*	Euroscarf
Δ*tkl1*	**BY4741**, Δ*tkl1*	Euroscarf
Δ*pck1*	**BY4741**, Δ*pck1*	Euroscarf
Δ*tpk1*	**BY4741**, Δ*tpk1*	Euroscarf
Δ*mig1*	**BY4741**, Δ*mig1*	Euroscarf
Δ*cat8*	**BY4741**, Δ*cat8*	Euroscarf
Δ*yck1*	**BY4741**, *Δyck1*	Euroscarf

The yeast deletion mutants used in this study were isogenic to one of the two parental strains, i.e., W303-1A [70] or BY4741. The wild type BY4741 strain and its deletion mutants were purchased from the EUROSCARF collection (www.uni-frankfurt.de/fb15/mikro/euroscarf).

*Escherichia coli* JM109 (*end*A1, *rec*A1, *gyr*A96, *thi*, *hsd*R17(r_k_^−^, m_k_^+^)*sup*E44, λ^−^, Δ(*lac* – *pro*AB), [F', *tra*D36, *pro*AB, *lac*I^q^ZM15] strain (Promega) was used for multiplication of plasmids.

### Media and growth conditions

*Saccharomyces cerevisiae* strains were cultured on YPD medium: 1% yeast extract (Becton, Dickinson & Company^®^), 2% peptone (Becton, Dickinson & Company^®^), 2% glucose (Chempur^®^), YP10 medium: 1% yeast extract (Becton, Dickinson & Company^®^), 2% peptone (Becton, Dickinson & Company^®^), 10% glucose (Chempur^®^) and N3 medium 1% yeast extract (Becton, Dickinson & Company^®^), 2% peptone (Becton, Dickinson & Company^®^), 2% glycerol (Chempur^®^). The spot-tests were performed using minimal synthetic (SD) medium with sucrose: 0.67% Yeast Nitrogen Base (Becton, Dickinson & Company^®^), 2% sucrose (Chempur^®^). The media were supplemented with uracil, adenine, histidine, leucine, tryptophane (Sigma^®^) at the concentration 10 μg/ml, when needed. Complete YPD medium with 200 μg/ml of geneticin (G418, Sigma-Aldrich) was used for selection of geneticin-resistant strains. To solidify the media 2% agar-agar was used (Becton, Dickinson & Company^®^). Yeast strains were grown at 28°C for 72, 96 and 120 hours, unless stated differently. Liquid cultures were grown overnight at 28°C and 160 rev./min [71].

*Escherichia coli* strain were cultured on LB medium: 1% bactotrypton (Becton, Dickinson & Company^®^), 1% yeast extract (Becton, Dickinson & Company^®^), 0,5% NaCl (Chempur^®^). When needed the medium was supplemented with ampicillin (Sigma-Aldrich) to the concentration of 100 μg/ml. To solidify the media 2% agar-agar was used (Becton, Dickinson & Company^®^). All bacterial strains were cultured at 37°C and 200 rev./min.

### Other chemicals and reagents

3-bromopyruvic acid, PEG and lithium acetate were purchased from Sigma-Aldrich (USA).

### Plasmids and primers

Plasmids and primers used in this study are presented in Tables 2 and 3 [72, 73]. Synthesis of primers was performed by Genomed (Warsaw, Poland).

Yeast genomic DNA library in the pFL44L plasmid was used (Lacroute, Gif-sur-Yvette).

Plasmid preparation, *E. coli* transformation and agarose gel electrophoresis were carried out as described in Sambrook and Russel [74].

Isolation of plasmid DNA was performed using standard alkaline lysis method or with Plasmid Mini Kit (A&A Biotechnology), according to the product manual.

### Spot-test method

To determine the MIC values (minimal inhibitory concentrations) of a tested compound toward the selected yeast strain and to compare the relative susceptibility of several strains, the cells were grown to mid-log phase, diluted to OD6_00_≈0.25 and spotted (3 μl) in 10-fold serial dilutions (10^0^, 10^−1^, 10^−2^) onto the agar plates containing various concentrations of a tested compound. Plates were incubated at 28°C and photographed after 72 h, 96 h or 120 h, depending when the phenotypic effect was most visible. The sensitivity assays were repeated a minimum of three times. Differences in growth show variability of the tested strain in their susceptibility to the tested inhibitor [34]. Spot tests were performed on minimal (YNB) medium with sucrose as a carbon source, unless stated differently.

### Determination of intracellular ATP levels in yeast cells

ATP level were determined for cells cultivated in liquid SD medium with sucrose at 28°C. The 24h cultures were diluted to optical density OD_600_ = 0.25 with fresh SD medium. The cultures were then incubated in medium with 1.8 mM and 3 mM 3-BP and as a control in medium without 3-BP. At the start-point and after 1, 2, 3, 5 hours of incubation, 50 μl of cell culture were lysed and the ATP level was determined using the ATPlite™ Luminescence Assay System (PerkinElmer) and PerkinElmer EnSpire® Multimode Plate Reader. ATP levels at each time-point were calculated as a percentage of the positive control (without 3-BP) and were recalculated per living cells. Viability of the cells at each time-point was determined by plating a 100 μl sample of culture (using appropriate culture dilutions if necessary) on YPD medium. Colonies were counted after 72h of incubation at 28°C. The experiment was repeated a minimum of three times.

### Rho^0^ generation

To obtain the rho^0^ respiratory deficient mutant the parental strain was incubated in liquid YP10 medium with ethidium bromide (30 ug/ml) for 24 hours at 28°C. After incubation cells were plated on the YPD medium. The mutants were identified by colony size and confirmed by the lack of growth on medium with glycerol (N3) [75].

### High efficiency yeast transformation

Yeast high efficiency and one-step transformation were performed by the lithium acetate procedure [76].

### Yeast colony PCR

A yeast colony taken from an agar plate was suspended in 0.02 M NaOH solution. The sample was then boiled for 5 minutes and cooled on ice. After short centrifugation the supernatant was used for the PCR amplification. PCR were performed using Taq DNA Polymerase (Thermo Scientific).

### Construction of yeast deletion-mutants

Deletion mutants in the W303-1A background were constructed using the method based on homologous integration of disruption cassettes described by Wach et al. [77]. The cassettes (KanMX6 and TRP1) were amplified by PCR with gene-specific primers using the following PCR program: 1:00 initial denaturation at 95°C, 30 cycles of 0:30 95°C, 0:30 65°C, 1:00 72°C and final extension of 5:00 at 72°C. The PCR product was then used for high-efficiency transformation of a specific yeast strain. Positive transformants were isolated using selective media: SD-trp for TRP1 cassette and YPD+G418 for KanMX6 cassette. To confirm successful deletion of chosen genes, total DNA was isolated from the transformed strain and control PCR reactions were performed with gene- and cassette-specific primers using the following PCR program: 1:00 initial denaturation at 95°C, 30 cycles of 0:30 95°C, 0:30 55°C, 1:00 72°C and final extension of 5:00 at 72°C. PCR were performed using Taq DNA Polymerase (Thermo Scientific).

### Search for multicopy suppressors of the resistant phenotype using yeast genomic DNA library

FY1679-28C was transformed with genomic DNA library (multicopy pFL44L plasmid) using high efficiency transformation, plated on SD medium without uracil (100 plates) and incubated for 48 hours. Grown transformants (200-300 colonies per plate) were then replicated on plates with SD medium containing 3 mM 3-BP and incubated for 120 hours. Total DNA was isolated from resistant transformants and used for transformation of *E.coli* JM109 to select and isolate plasmid DNA from total DNA. Isolated plasmids were sequenced using M13FWD and M13REV primers (Genomed, Warsaw, Poland) and used for retransformation of yeast, to confirm the resistant phenotype.

### Statistical analysis

The results represent the mean ± SD from at least three independent experiments. Statistical significance was assessed by 1-way ANOVA using GraphPad Prism5, and with Tukey's multiple comparison test.
